# Socioeconomic Status Across the Life Course and Old Age Cognition

**DOI:** 10.1093/geroni/igaf122.2298

**Published:** 2025-12-31

**Authors:** Michael Topping

**Affiliations:** University of Wisconsin–Madison, Madison, Wisconsin, United States

## Abstract

While linkages between socioeconomic conditions and cognition are well-documented, how socioeconomic conditions operate across the whole life course as a fundamental cause of disparities in cognition is less clear. Furthermore, many prior studies disproportionately rely on retrospective reports of socioeconomic status from earlier in life, and also utilize unitary, rather than multidimensional constructs of socioeconomic status. Drawing on rich prospective data from the Wisconsin Longitudinal Study over six decades, I constructed latent measures of socioeconomic status for individuals for the entire cohort across key life course periods: early life, adolescence, early adulthood, mid-life and later life, to examine both the independent and joint impact of socioeconomic status on cognition in old age. Results from linear regression analyses show that socioeconomic status at each life course period has a positive impact on cognition at age 80. However, upon modeling all life course periods, all are completely mediated, save for those corresponding to early and later life, which are substantially reduced. This implies that the effects of early life socioeconomic status operate directly and indirectly across the life course to shape global cognitive functioning. Additional results using sequence analysis revealed those who had trajectories of downward mobility or those who faced persistent, prolonged exposure to low socioeconomic status across the life course had the lowest levels of cognition, relative to those who experienced continuous socioeconomic advantage throughout their lifetimes. These findings have profound implications for estimating the impact of how socioeconomic conditions operate independently and in-tandem to shape later life cognition.

